# GPS Cycle Slip Detection Considering Satellite Geometry Based on TDCP/INS Integrated Navigation

**DOI:** 10.3390/s151025336

**Published:** 2015-09-30

**Authors:** Younsil Kim, Junesol Song, Changdon Kee, Byungwoon Park

**Affiliations:** 1 Institute of Advanced Aerospace Technology, School of Mechanical and Aerospace Engineering, Seoul National University, 1 Gwanak-ro, Gwanak-gu, Seoul 151-744, Korea; E-Mails: younsil@snu.ac.kr (Y.K.); june85@snu.ac.kr (J.S.); 2 School of Mechanical and Aerospace Engineering, Sejong University, 209 Neungdong-ro, Gwangjin-gu, Seoul 143-747, Korea; E-Mail: byungwoon@sejong.ac.kr

**Keywords:** cycle slips, inertial sensors, TDCP/INS integrated system

## Abstract

This paper presents a means of carrier phase cycle slip detection for an inertial-aided global positioning system (GPS), which is based on consideration of the satellite geometry. An integrated navigation solution incorporating a tightly coupled time differenced carrier phase (TDCP) and inertial navigation system (INS) is used to detect cycle slips. Cycle-slips are detected by comparing the satellite-difference (SD) and time-difference (TD) carrier phase measurements obtained from the GPS satellites with the range estimated by the integrated navigation solution. Additionally the satellite geometry information effectively improves the range estimation performance without a hardware upgrade. And the covariance obtained from the TDCP/INS filter is used to compute the threshold for determining cycle slip occurrence. A simulation and the results of a vehicle-based experiment verify the cycle slip detection performance of the proposed algorithm.

## 1. Introduction

Currently, meter-level positioning accuracy is easily achieved using pseudorange measurements made with GPS satellites and is widely used in many types of industry. However, for centimeter-level positioning accuracy, the pseudorange measurements are not sufficient because of the high levels of noise. Therefore, carrier-phase-based positioning is necessary to achieve centimeter-level accuracy, such as Real Time Kinematic (RTK) navigation. Currently, carrier-phase based positioning is being widely studied for application to services demanding high-accuracy positioning, including the navigation of aircraft and land vehicles.

However, when using the carrier-phase as a positioning measurement, carrier-phase fault detection must be performed before the positioning. Cycle slip detection is one of the most important issues to be overcome when implementing a GPS carrier phase positioning system, because cycle slip occurs very frequently whenever the carrier phase signal is weak. This results in the degradation of the vehicle’s positioning accuracy.

Cycle slip detection algorithms have been studied and developed for several decades. The phase-code comparison, phase-phase ionospheric residual, doppler integration, and differential phases of time methods have been used to detect cycle slips [1]. For a dual-frequency GPS receiver, Bisnath *et al.*, Blewitt, and Gao *et al*. proposed cycle slip detection based on L1 and L2 carrier phase measurements [2,3,4,5]. Recently, Banville and Langley *et al*. suggested algorithms that are not affected by high ionospheric activity [6,7,8,9]. These algorithms were basically developed for dual frequencies, such that they can be applied only to a multiple-frequency receiver. Consequently, compared to a single-frequency receiver, the overall system cost would be high and therefore not cost effective. For a single-frequency GPS receiver, the phase-code comparison, doppler integration, and differential phases of time methods can be used. However, because of the high noise level of the code measurement the phase-code comparison method can only safely handle a few cycle slips. The doppler integration method and the differential phases of time method also have a limited cycle slip detection accuracy, especially in dynamic environments, due to the low data rates of GPS receivers [10,11].

To overcome these limitations, an inertial navigation system (INS) can be integrated with a GPS to detect cycle slips. This method can be implemented with a single-frequency receiver. Altmayer, Colombo *et al*. used GPS/INS integrated systems to detect cycle slips [12,13,14,15,16]. The INS can find the vehicle’s current position by integrating its rate information from the previous position. The distance from the satellite to the INS-determined current position is equivalent to the measured carrier phase, so that the cycle-slip can be detected by comparing the two values. In this algorithm, the accuracy of the INS position estimation is the main contributor to the cycle slip detection. In turn, the accuracy of the inertial sensor is the most important contributor to the performance of an INS system. Therefore, if the inertial sensor is accurate enough, one-cycle slip detection is achievable. However, the accuracy of an inertial sensor is directly proportional to its cost, such that the most accurate devices are prohibitively expensive.

Therefore, in this paper, we discuss how to improve the performance of the inertial-aided cycle slip detection algorithm, such that it can detect one-cycle slip, without having to increase the inertial sensor accuracy. The SD and TD residual between the predicted and measured carrier phases is defined as the value to be monitored for cycle slip detection. As the value of the residual decreases, the cycle slip detection accuracy increases. The INS position error mainly contributes to the residual and is projected to the range domain, multiplied by the SD line of sight vector. In general, the satellite having the highest elevation angle is chosen as the reference satellite for SD, with the same applying to all other satellites when obtaining SD. However, by selecting the SD satellite pair based on the satellite geometry, which minimizes the INS position error projection to the range domain, the cycle slip detection accuracy can be advanced with the same inertial sensor performance. In the proposed algorithm, the reference satellite is selected as the nearest satellite and therefore the reference satellite usually differs depending on the satellite. To construct independent satellite pairs, a tree structure is utilized [17]. Then, cycle slips as obtained from a general satellite pair can be reconstructed by using an invertible transformation matrix with no loss of information from the cycle slips detected using the proposed satellite pair.

In the proposed algorithm, the tightly coupled TDCP/INS integrated navigation algorithm is used to estimate the user position. Before the TDCP measurement is updated, cycle slip detection and isolation is conducted by using the INS-predicted state variable. The cycle slip detection threshold is calculated by using the INS predicted covariance to maintain a consistent cycle slip false alarm probability. After cycle slip detection and isolation, the remaining carrier phase measurements are inserted as measurements of the TDCP/INS integrated navigation for the cycle slip detection for the next epoch.

To verify the cycle slip detection performance for the proposed algorithm, a simulation and a vehicle-based experiment were conducted. Data was collected from the single-frequency GPS receiver, microelectromechanical systems (MEMS) inertial measurement units (IMU) for post processing. We analyzed the cycle-slip detection performance by statistical analysis. As a result, the proposed satellite pair selection algorithm improves the cycle slip detection probability considerably.

## 2. Cycle Slip Detection Algorithm

### 2.1. Monitoring Value for Cycle Slip Detection

To detect cycle slips, the SD and TD residual between the measured and predicted carrier phases is defined as the monitoring value for cycle slip detection. Unlike conventional cycle slip detection algorithms that use linear combinations of multiple-frequency GPS observations, this cycle slip detection algorithm uses only single-frequency GPS observations. Therefore, the proposed algorithm can detect cycle slips in all situations, regardless of the sizes of the simultaneous cycle slips that occur at multiple frequencies.

To derive the monitoring value, we take the SD and TD of the carrier phase measurement. In this process, the clock bias terms, atmosphere-related errors, and orbit errors are greatly reduced [18,19].

Then, the resulting monitoring value can be defined as follows:

(1) $$M_{k+1}^{i}{=}^{i}\nabla^{j}{\text{Δ}}_{t}{\text{ϕ}}_{GPS}{-}^{i}\nabla^{j}{\text{Δ}}_{t}{\text{ϕ}}_{INS}$$

The subscript GPS represents the value obtained from the GPS receiver while the subscript INS indicates the value computed from the INS. Furthermore, the superscript *i* is the satellite index, and the subscript *k* is the GPS epoch index. The ^*i*^∇^*j*^ is the SD operator between the satellite *i* and *j* and the ${\text{Δ}}_{t}$ is the TD operator between the GPS epochs.

The prediction of the carrier phase term in Equation (1) is performed by using INS. The INS estimates the position, velocity, and attitude of the vehicle. To detect cycle slip in the *k* + 1 epoch, the inertial sensor measurements between the *k* and *k* + 1 epochs, velocity, and attitude in the *k* epoch are used.

SD carrier phase measurements are considered instead of single raw carrier phase measurements because most RTK algorithms are usually based on SD phase measurements to compensate for the GPS receiver clock bias error [12]. Hence, the cycle slips detected by the monitoring value imply differenced values in this paper.

Then, by calculating the monitoring value for each epoch, cycle slips can be detected, as follows:

$$M_{k+1}^{i}\left\{ \begin{array}{l} <threshold\;\to \text{ no cycle slip}\\ \geq threshold\;\to \text{ cycle slip} \end{array}\right.$$

The threshold for the monitoring differs from the required cycle slip detection performance. In this algorithm, the cycle slip detection threshold is determined by using the INS-predicted covariance to maintain the consistent cycle slip false alarm probability.

#### 2.1.1. Monitoring Value Error Analysis

To analyze the contribution of the error sources to the monitoring value, an error analysis was performed, as follows:

(2) Mk+1i=(∇jiΔtϕGPS−i∇jΔtϕ)−(∇jiΔtϕINS−i∇jΔtϕ)

or:

(3) $$M_{k+1}^{i}={\text{δ}}^{i}\nabla^{j}{\text{Δ}}_{t}{\text{ϕ}}_{GPS}-{\text{δ}}^{i}\nabla^{j}{\text{Δ}}_{t}{\text{ϕ}}_{INS}$$

The ϕ term is the true value of the carrier phase and is the sum of the integer ambiguity and the distance between the satellite and the user. Furthermore, δ implies an error value.

**Figure 1:**
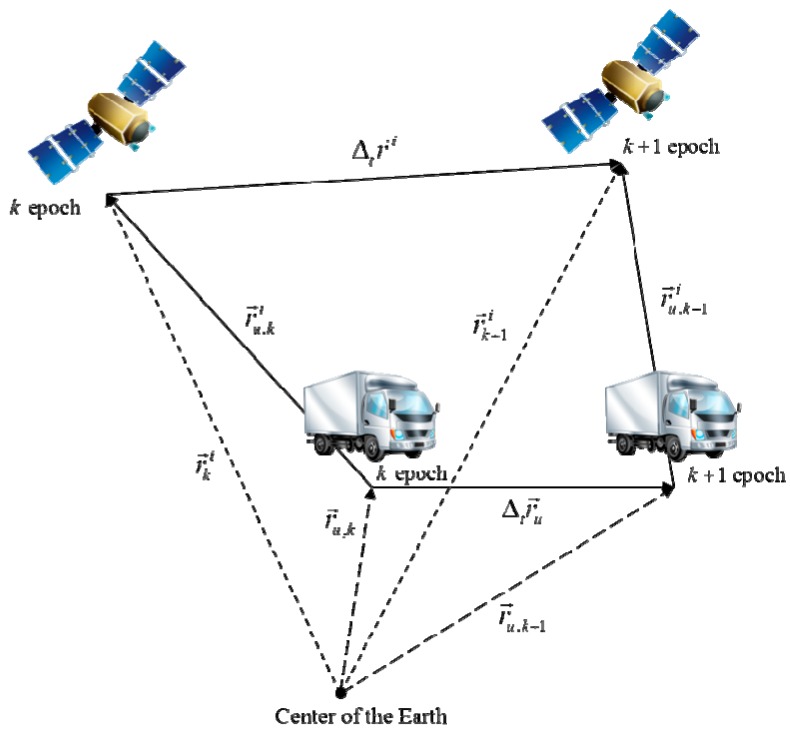
User and satellite configuration during consecutive GPS epochs.

Before further derivation is performed, the definitions of the symbols are represented graphically. Figure 1 presents the user and satellite configuration during consecutive GPS epochs. Each of the terms represented in Figure 1 is used to express the error equation for the monitoring value. 

By using a graphical representation of the user and satellite geometry, the true value of the different carrier phases in Equation (2) can be expressed, as follows. The arrow represent the vector quantity. And ${\vec{e}}_{k}^{i}$ represents the line of sight vector of *i*-th satellite at *k* epoch:

(4) ∇jiΔtϕ=|Δtr→ui|−|Δtr→uj|

In more detail:

(5) ∇jiΔtϕ=(r→k+1i−r→u,k+1)⋅e→k+1i−(r→ki−r→u,k)⋅e→ki−(r→k+1j−r→u,k+1)⋅e→k+1j+(r→kj−r→u,k)⋅e→kj

Then, we analyze the errors in Equation (3), term-by-term. First, the measurement error in the GPS carrier phase is investigated:

(6) $${\text{δ}}^{i}\nabla^{j}{\text{Δ}}_{t}{\text{ϕ}}_{GPS}\approx^{i}\nabla^{j}{\text{Δ}}_{t}N\text{λ}{+}^{i}\nabla^{j}{\text{Δ}}_{t}\text{ε}$$

Referring to the reference paper, the clock and atmosphere-related terms can be eliminated after the differencing process [18,19]. Then, the cycle slip term ∇jiΔtNλ and the carrier phase noise term ∇jiΔtε remain after the time and satellite difference.

Next, the error in the predicted carrier phase arising from the INS is investigated. Based on the results of our previous study, we can derive the INS-predicted carrier phase error, as follows [19]:

(7) $${\text{δ}}^{i}\nabla^{j}{\text{Δ}}_{t}{\text{ϕ}}_{INS}\approx {-}^{i}\nabla^{j}{\vec{e}}_{k}\cdot \text{δ}{\text{Δ}}_{t}{\vec{r}}_{u}$$

The term $\text{δ}{\text{Δ}}_{t}{\vec{r}}_{u}$ represents the relative position estimation error between the GPS epochs, while ∇jie→k is the computed SD line of sight vector for the *k* epoch. From Equation (7), it can be seen that the monitoring value error is mainly affected by the relative position estimation error between the epochs, not the absolute position error. Therefore, the INS can be used to estimate the relative position between the GPS data epochs. This is because the INS has a good positioning accuracy when the integration time interval is short. In this case, the integration time interval is less than 1 s.

By combining Equations (6) and (7), the monitoring value equation can be represented by Equation (8):

(8) $$M_{k+1}^{i}{=}^{i}\nabla^{j}{\text{Δ}}_{t}N\text{λ}{+}^{i}\nabla^{j}{\text{Δ}}_{t}\text{ε}{+}^{i}\nabla^{j}{\vec{e}}_{k}\cdot \text{δ}{\text{Δ}}_{t}{\vec{r}}_{u}$$

Considering the ideal case, the monitoring value represents the degree of cycle slip. Then, we can define the monitoring value error by Equation (9):

(9) $$\text{δ}M_{k+1}^{i}{=}^{i}\nabla^{j}{\text{Δ}}_{t}\text{ε}{+}^{i}\nabla^{j}{\vec{e}}_{k}\cdot \text{δ}{\text{Δ}}_{t}{\vec{r}}_{u}$$

In Equation (9), the carrier phase noise term is negligible because the standard deviation of the GPS L1 carrier phase measurement is conservatively about 3 mm and is much smaller than the INS position error term [20]:

(10) $$\text{δ}M_{k+1}^{i}\approx^{i}\nabla^{j}{\vec{e}}_{k}\cdot \text{δ}{\text{Δ}}_{t}{\vec{r}}_{u}$$

Then, the relative position estimation error between the consecutive GPS epochs determines the cycle slip detection performance, as defined by Equation (10). If the position estimation error term is sufficiently small, we can precisely detect the cycle slip. If, however, the position error is large, we cannot distinguish the cycle slip from the monitoring value because the cycle slip is concealed from the position error.

Equation (10) also indicates that the relative position error is projected onto the range domain multiplied by the SD line of sight vector. In general, the satellite having the highest elevation angle is used as the reference satellite for SD because of the low noise level of the carrier phase measurement relative to the other satellites. In this algorithm, however, the relative position estimation error has a more significant effect than the carrier phase measurement error on the monitoring value error. Therefore, even if the carrier phase measurement noise is larger for a satellite at a lower elevation angle, it is preferable to select the nearest satellite as the reference satellite, rather than the satellite with the highest elevation angle. This was verified by the results of our experiments.

### 2.2. Applying Satellite Geometry to Cycle Slip Detection

Based on the motivation described in the previous section, we applied the satellite geometry to the inertial-aided cycle slip detection.

The vector form of Equation (10) can be written as follows for a general SD. The *j*-th satellite is used as a reference satellite and has the highest elevation angle in the line of sight:

(11) [δMk+11δMk+12⋮δMk+1n−1]=[∇j1e→kT∇j2e→kT⋮∇jn−1e→kT]δΔtr→u

Then, the relative position error projection coefficient matrix to the range domain can be defined by Equation (12):

(12) C=[∇j1e→kT∇j2e→kT⋮∇jn−1e→kT]

The detected cycle slips based on this monitoring value are represented as follows:

(13) N→=[∇j1ΔtN∇j2ΔtN⋮∇jn−1ΔtN]

However, by selecting the nearest satellite as a reference for each satellite, we can reduce the magnitude of the projection coefficient of the relative position estimation error.

Equation (14) represents the projection coefficient matrix by considering the satellite geometry, while Equation (15) represents the cycle slips according to each satellite pair. To reduce the projection coefficient, the satellite pair for SD should be selected to minimize the distance between the two satellites:

(14) C'=[∇i1e→kT∇j2e→kT⋮∇kn−1e→kT]

(15) N→'=[∇i1ΔtN∇j2ΔtN⋮∇kn−1ΔtN]

The cycle slips based on the general SD pair can be reconstructed by using the invertible transformation matrix *T*, as follows:

(16) $$\begin{array}{l} \vec{N}=T\;{\vec{N}}^{'}\\ {\vec{N}}^{'}=T^{-1}\vec{N} \end{array}$$

To establish the invertible transformation matrix *T*, it is necessary to construct proper satellite pairs for which there is no dependent pair. For a total of *n* satellites, there is *n* − 1 independent SD pairs. To attain independence, the combination of *n* satellites should have a tree structure. A tree structure offers a means of representing the hierarchical nature of a structure in a graphical form, which is useful when building independent SD pairs. The process of satellite pairing is explained in the next section.

#### 2.2.1. Satellite Pairing

**Figure 2:**
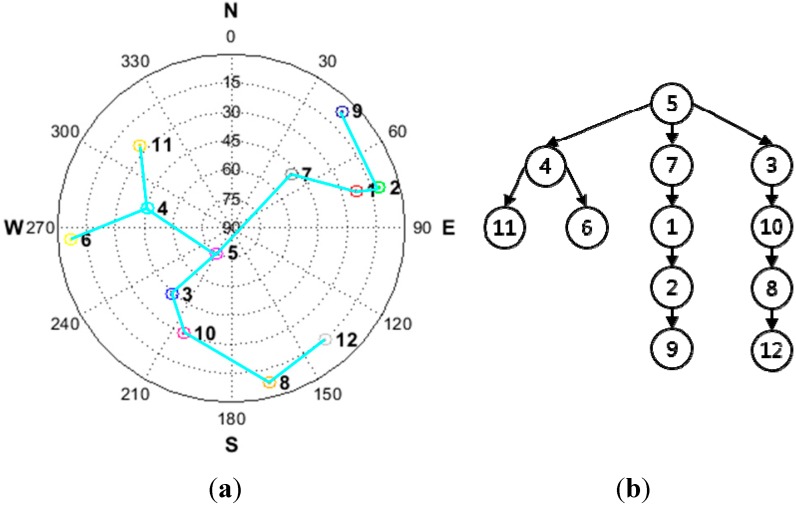
(**a**) Example satellite geometry; (**b**) resulting tree structure.

Figure 2 shows an example of the satellite construction results based on a given satellite geometry. It is obvious that the resulting satellite pair structure is a tree structure. The properties of a typical tree structure are such that the structure has no closed loop. If a closed loop were to occur, it would give rise to linear dependency and this would cause the transformation matrix *T* to be non-invertible.

In Figure 2b, the point represented by the satellite’s pseudo random number (PRN) is called a “node.” A connection between at least two nodes constitutes a branch. We refer to a connection between two nodes a “single branch.” Each single branch represents the proposed satellite pair at SD. The root node, allocated at the top of the tree structure, is the reference satellite for the general SD with the highest elevation angle.

First, to construct the proposed satellite pair, the norm for all of the possible combinations of the SD line of sight vector is computed as follows. The resulting matrix *E* is the upper triangular matrix, consisting of n(n−1)/2 non-zero components:

(17) E=[0|∇21e→|⋯|∇n1e→|⋮0⋮0⋱|∇nn−1e→|00…0]

Second, all of the non-zero components in matrix *E* are sorted into ascending order, as given by Equation (18):

(18) $$\vec{E}={\left[\begin{array}{ccc} {\left|\nabla \vec{e}\right|}_{\min} & \cdots & {\left|\nabla \vec{e}\right|}_{\max} \end{array}\right]}^{T}$$

**Figure 3:**
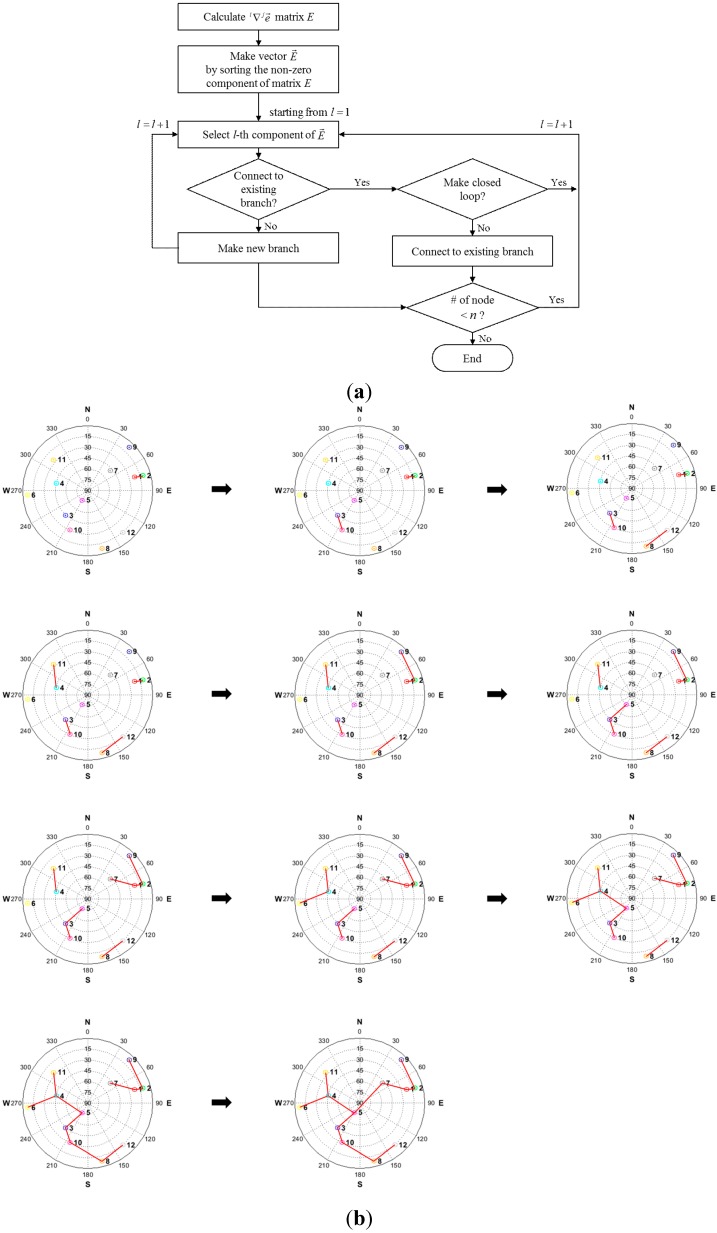
(**a**) Block diagram of satellite pairing process (above); (**b**) sequence of satellite pairing process (below).

Then, starting from the first element of vector $\vec{E}$, the proposed satellite pair structure is formed through iterating the process shown in Figure 3a. Figure 3 shows the satellite pairing process. If a current satellite pair makes a new branch, the new branch is built. Alternatively, if the current satellite pair is connected to an existing branch, a check should first be made as to whether the new branch forms a closed loop. When the number of nodes exceeds *n*, the process is ended. In the process, a few single branches are first made and then the number of nodes in the existing branches is increased by getting a new node or by connecting to the other branch. This algorithm is very simple and effective. Because the satellite geometry varies slowly, the satellite pair does not have to be calculated every second. Rather, the calculation interval can be as long as several minutes. In addition, the satellite pairs have to be updated only when a new satellite rises or an existing satellite disappears. Therefore, the computational load incurred by the satellite-pairing algorithm is minimal.

#### 2.2.2. Transformation Matrix for Reconstruction

Once the tree structure is constructed, the conversion of the detected cycle slips from the proposed SD pair to the general SD pair is possible by using Equation (16). This is done by using the transformation matrix *T*. The matrix *T* can be calculated from the resulting tree structure. 

Equation (19) shows the cycle slips in the proposed SD pair. The satellite PRN 5 is the reference satellite of the general SD pair:

(19) N→'=[∇71ΔtN∇12ΔtN∇53ΔtN∇54ΔtN∇46ΔtN∇57ΔtN∇108ΔtN∇29ΔtN∇310ΔtN∇411ΔtN∇812ΔtN]

Given the resulting tree structure, the cycle slips in the general SD pair can be computed as follows. For example, for satellite PRN 1, satellite PRN 7 is selected as a reference satellite. To establish the connection between satellite PRN 1 and satellite PRN 5, satellite PRN 7 is needed to perform the role of the bridge, as shown in Figure 2b. By using this connection, the transformation from the proposed SD pair to the general SD pair can be performed. Equations (20) and (21) show this transformation:

(20) ∇51ΔtN=1∇7ΔtN+7∇5ΔtN

or:

(21) ∇51ΔtN=(ΔtN1−ΔtN7)+(ΔtN7−ΔtN5)

Then the transformation matrix *T* can be calculated starting from the identity matrix by assigning one to the space of the bridge satellites in matrix *T*, as explained above. For example, in the first row of the transformation matrix in Equation (22), one is assigned to the space for satellite PRN 7. The remaining rows are calculated in the same way.

Equation (22) shows the transformation matrix for the example.

(22) $$T=\left[\begin{array}{ccccccccccc} 1 & 0 & 0 & 0 & 0 & 1 & 0 & 0 & 0 & 0 & 0\\ 1 & 1 & 0 & 0 & 0 & 1 & 0 & 0 & 0 & 0 & 0\\ 0 & 0 & 1 & 0 & 0 & 0 & 0 & 0 & 0 & 0 & 0\\ 0 & 0 & 0 & 1 & 0 & 0 & 0 & 0 & 0 & 0 & 0\\ 0 & 0 & 0 & 1 & 1 & 0 & 0 & 0 & 0 & 0 & 0\\ 0 & 0 & 0 & 0 & 0 & 1 & 0 & 0 & 0 & 0 & 0\\ 0 & 0 & 1 & 0 & 0 & 0 & 1 & 0 & 1 & 0 & 0\\ 1 & 1 & 0 & 0 & 0 & 1 & 0 & 1 & 0 & 0 & 0\\ 0 & 0 & 1 & 0 & 0 & 0 & 0 & 0 & 1 & 0 & 0\\ 0 & 0 & 0 & 1 & 0 & 0 & 0 & 0 & 0 & 1 & 0\\ 0 & 0 & 1 & 0 & 0 & 0 & 1 & 0 & 1 & 0 & 1 \end{array}\right]$$

The transformation matrix *T* always has full rank because of the properties of a tree structure. This means that the detected cycle slips can be fully reconstructed with no loss of information in either direction.

### 2.3. Cycle Slip Detection Performance Index and Threshold

This section explains the cycle slip detection performance index and threshold determination and the computed threshold cycle slip occurrence discrimination.

**Figure 4:**
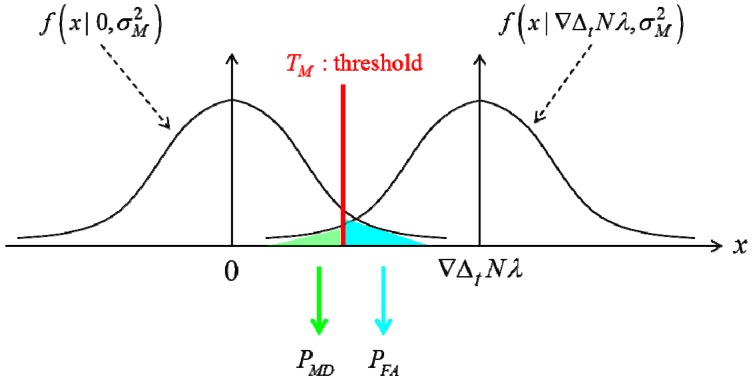
Monitoring value probability distribution function.

Figure 4 shows the probability distribution for the monitoring value for the cycle slip occurrence case and the opposite case.

When no cycle slip occurs, the probability distribution function can be written as follows. Equation (23) represents the Gaussian probability density function with a zero-mean and the variance with ${\text{σ}}_{M}^{2}$:

(23) $$f\left(x|0,{\text{σ}}_{M}^{2}\right)=\frac{1}{\sqrt{2\pi }{\text{σ}}_{M}}\exp\left[-x^{2}/2{\text{σ}}_{M}^{2}\right]$$

When a cycle slip occurs, the probability distribution function is shifted by an amount equal to the cycle slip $\nabla {\text{Δ}}_{t}N\text{λ}$. The SD satellite index is neglected for simplicity:

(24) $$f\left(x|\nabla {\text{Δ}}_{t}N\text{λ},{\text{σ}}_{M}^{2}\right)=\frac{1}{\sqrt{2\pi }{\text{σ}}_{M}}\exp\left[-{\left(x-\nabla {\text{Δ}}_{t}N\text{λ}\right)}^{2}/2{\text{σ}}_{M}^{2}\right]$$

In Figure 4, given the threshold, the monitoring value standard deviation determines the false alarm and miss detection probabilities. These two probabilities are the performance index of the cycle slip detection algorithm.

The false alarm and miss detection probabilities can thus be calculated by using Equation (25) [1,15,19]:

(25) $$\left\{ \begin{array}{l} P_{FA}=2\int_{T}^{\infty }f\left(x|0,{\text{σ}}_{M}^{2}\right)dx\\ P_{MD}=2\int_{T}^{\infty }f\left(x|\nabla {\text{Δ}}_{t}N\text{λ},{\text{σ}}_{M}^{2}\right)dx \end{array}\right.$$

The main contribution of the proposed SD method is the improvement of the cycle slip detection performance index by reducing the standard deviation of the monitoring value.

**Figure 5:**
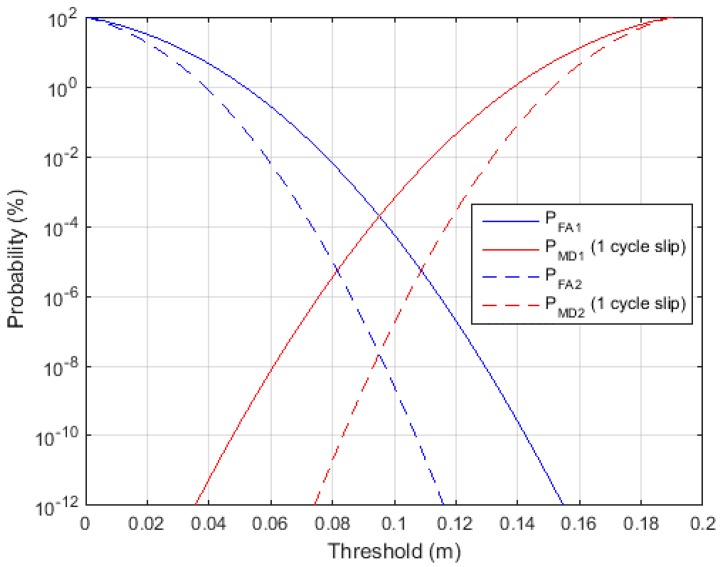
Probability of false alarm and miss detection.

More precisely, Figure 5 shows the effect of the monitoring value standard deviation on the cycle slip performance. In Figure 5, subscript 1 in the legend indicates the value of ${\text{σ}}_{M1}$ which is represented as a line, while subscript 2 is the value of ${\text{σ}}_{M2}$, as represented using a dashed line. Furthermore, ${\text{σ}}_{M1}>{\text{σ}}_{M2}$. It is notable that the reduction in the standard deviation of the monitoring value improves both the false alarm and miss detection probability, unlike the threshold which only affects to the interrelationship between the two probabilities. Therefore, the reduction in the standard deviation of the monitoring value is very important to cycle slip detection.

The variance in the monitoring value can be calculated by using the INS-predicted state covariance and measurement noise, from Equation (9), as follows:

(26) var(Mk+1i)=var(∇jie→k⋅δΔtr→u)+var(∇jiΔtε)

In Equation (26), the covariance of the relative position estimation error can be calculated as follows. In the proposed algorithm, the standard deviation of the monitoring value is reduced by minimizing the projection coefficient, ∇jie→k, which either enlarges or shrinks the relative positioning error to the range domain.

First, the INS-predicted user position at the *k* + 1 epoch can be written as Equation (27):

(27) $${\vec{r}}_{u,k+1}^{-}={\vec{r}}_{u,k}^{+}+{\text{Δ}}_{t}{\vec{r}}_{u}$$

The superscript plus sign (+) represents the state variable after the TDCP measurement update in the Kalman filter, while the superscript minus sign indicates the state variable before the TDCP measurement update in that epoch. Therefore, the ${\vec{r}}_{u,k+1}^{-}$ represents the position vector at the *k* + 1 epoch which is propagated from the result of the *k* epoch by using the time update process.

In Equation (27), random vectors ${\vec{r}}_{u,k}^{+}$ and ${\text{Δ}}_{t}{\vec{r}}_{u}$ are independent because the relative position estimation vector depends only on the velocity and attitude in the *k* epoch. Therefore, the covariance of the sum of the position vector in the *k* epoch and the relative position vector can be represented as the sum of each covariance.

Therefore, the variance in the monitoring value can be calculated by using Equation (28):

(28) var(Mk+1i)=∑[(∇jie→k∇jie→kT)⊗{cov(r→u,k+1−)−cov(r→u,k+)}]+var(∇jiΔtε)

In Equation (28), the operator ⊗ represents the component product between matrixes of the same size, while ∑[⋅] represents the sum of all the components of the matrix in the square bracket.

By using the calculated variance of the monitoring value, the cycle slip detection threshold is determined. For this study, we selected a threshold that causes the miss detection probability to be 6.3342 × 10^−5^ for a one-cycle slip because, in statistics, the miss detection case is more critical. It can thus be adjusted according to the user’s preferences according to Figure 5. Furthermore, the threshold can be determined based on the required false alarm probability.

A threshold having a miss detection probability of 6.3342 × 10−5 for one-cycle slip detection can be calculated by setting $k_{M}$ to 4 in Equation (29):

(29) $$T_{M}^{i}=\text{λ}-k_{M}\cdot \sqrt{\mathrm{var}\left(M_{k+1}^{i}\right)}$$

## 3. Tightly Coupled TDCP/INS Integration Algorithm

**Figure 6:**
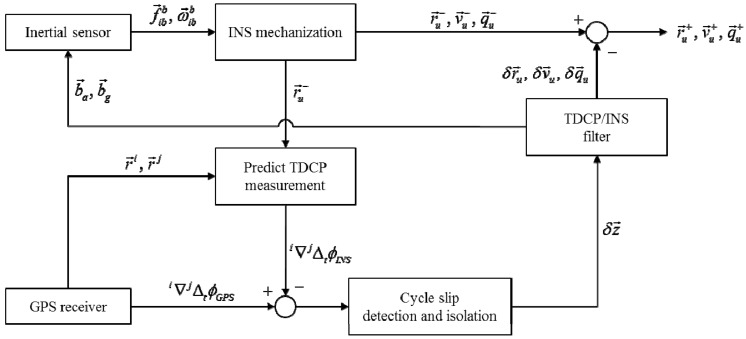
Tightly coupled TDCP/INS integration.

In the proposed algorithm, the tightly coupled TDCP/INS integrated navigation algorithm is used to estimate the user position. Figure 6 is a block diagram of the algorithm.

By using the inertial sensor output, the general INS mechanization predicts the user position and then, before the TDCP measurement update, cycle slip detection and isolation are conducted based on the INS predicted state. After the cycle slip detection and isolation, the remaining carrier phase measurements are inserted as measurements for the TDCP/INS-integrated navigation for cycle slip detection in the next epoch.

The INS mechanization is a nonlinear function of the state variable, with the detailed nonlinear equation referenced at [21,22,23]:

(30) $$\dot{\vec{x}}=fn\;\left(\vec{x}\right)$$

(31) $$\vec{x}={\left[\begin{array}{ccccc} {\vec{r}}_{u} & {\vec{v}}_{u} & {\vec{q}}_{u} & {\vec{b}}_{a} & {\vec{b}}_{g} \end{array}\right]}^{T}$$

In Equation (31), the states are user position, velocity, attitude, accelerometer bias, and gyro bias, in that order.

For the tightly coupled TDCP/INS filter, the state equation is presented as follows, with the detailed components of each matrix being referenced at [21,22,23]. Because we use the SD carrier phase as a measurement, the GPS clock bias and bias drift terms are neglected, unlike in the case of the general tightly coupled integration system:

(32) $$\text{δ}\dot{\vec{x}}=F\text{ δ}\vec{x}+G\;\vec{w}$$

(33) $$\text{δ}\vec{x}={\left[\begin{array}{ccccc} \text{δ}{\vec{r}}_{u} & \text{δ}{\vec{v}}_{u} & \text{δ}{\vec{q}}_{u} & \text{δ}{\vec{b}}_{a} & \text{δ}{\vec{b}}_{g} \end{array}\right]}^{T}$$

After the INS prediction, the TDCP measurement is preprocessed by using the predicted TDCP. If the monitoring value exceeds the threshold, cycle slip is detected. Furthermore, the measurements which have a cycle slip are isolated, and then the remaining measurements are used.

The measurement model for the TDCP/INS filter can be derived as Equation (34) by using the TDCP measurement. Originally, based on the TDCP measurement model, the current measurement is connected linearly to the previous state variables, which violates the usual format of the Kalman filter model [24,25]. Therefore, for simplicity, we use a simplified measurement model with some modifications [20]:

(34) $$\text{δ}z_{k+1}^{i}{=}^{i}\nabla^{j}{\text{Δ}}_{t}\tilde{\text{ϕ}}-\left\{\left(\left|{\vec{r}}_{k+1}^{i}-{\vec{r}}_{u,k+1}^{-}\right|-\left|{\vec{r}}_{k}^{i}-{\vec{r}}_{u,k}^{+}\right|\right)-\left(\left|{\vec{r}}_{k+1}^{j}-{\vec{r}}_{u,k+1}^{-}\right|-\left|{\vec{r}}_{k}^{j}-{\vec{r}}_{u,k}^{+}\right|\right)\right\}$$

(35) $$\text{δ}z_{k+1}^{i}={-}^{i}\nabla^{j}{\vec{e}}_{k+1}^{T}\text{ δ}{\vec{r}}_{u,k+1}+v^{i}$$

Finally, the measurement is updated for cycle slip detection in the next epoch by using Equation (36):

(36) $$\text{δ}{\vec{z}}_{k+1}=H_{k+1}\text{ δ}{\vec{x}}_{u,k+1}+\vec{v}$$

(37) $$H_{k+1}=\left[\begin{array}{ccccc} {-}^{i}\nabla^{j}{\vec{e}}_{k+1}^{T} & 0_{3\times 1} & 0_{3\times 1} & 0_{3\times 1} & 0_{3\times 1}\\ \vdots & \vdots & \vdots & \vdots & \vdots \\ {-}^{l}\nabla^{j}{\vec{e}}_{k+1}^{T} & 0_{3\times 1} & 0_{3\times 1} & 0_{3\times 1} & 0_{3\times 1} \end{array}\right]$$

## 4. Simulation and Experimental Results

To investigate the cycle slip detection performance of the proposed algorithm, a simulation and vehicle-based experiment were conducted. Because the proposed algorithm was developed for application to the preprocessing of carrier-phase-based positioning, the simulation and experiment addressed environments with more than four visible satellites. It is impossible to derive a positioning solution with carrier phase measurement when there are fewer than four visible satellites.

### 4.1. Simulation Results

#### 4.1.1. Simulation Environments

To verify the proposed algorithm, simulation data was generated for a trajectory with mixed maneuvers, driving at a constant speed, forward acceleration, and turning at different rates of rotation. Inertial sensor data is generated according to the commercial MEMS IMU data specification [26].

The GPS simulation data was generated for the errors listed in Table 1. The GPS output rate was set to 1 Hz.

**Table 1:** GPS simulation generation method.

GPS Errors	Generation Strategy
Ephemeris error	Neglect
Ionospheric delay	Klobuchar model [27]
Tropospheric delay	Simplified model [28]
Receiver noise	Zero-mean Gaussian noise (σ = 3mm)

IMU simulation data were generated for the errors listed in Table 2. The IMU output rate was set to 100 Hz.

**Table 2:** IMU simulation generation method.

GPS Errors	Generation Strategy
Accelerometer bias	Constant bias (0.1635 m/s^2^)
Gyro bias	Constant bias (1 °/s)
Accelerometer noise	Zero-mean Gaussian noise (σ = 0.0333 m/s^2^)
Gyro noise	Zero-mean Gaussian noise (σ = 0.0333 °/s)

**Figure 7:**
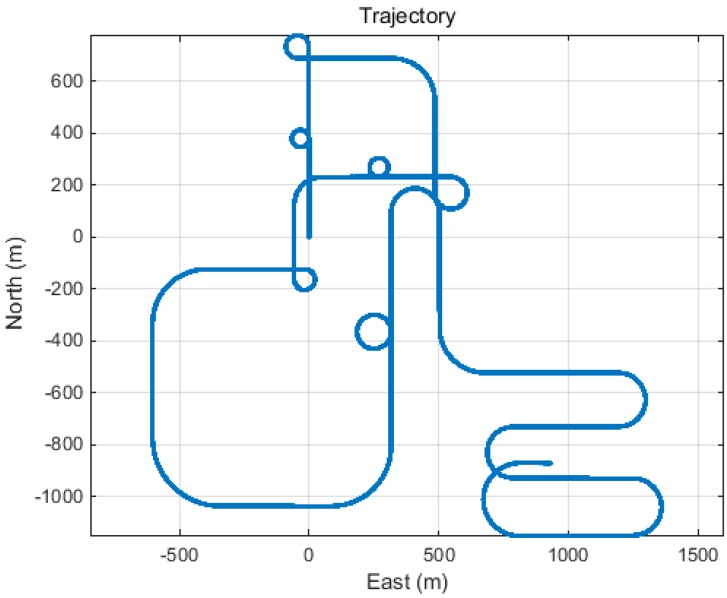
Generated user trajectory.

Figure 7 shows the generated trajectory for a mixture of maneuvers.

**Figure 8:**
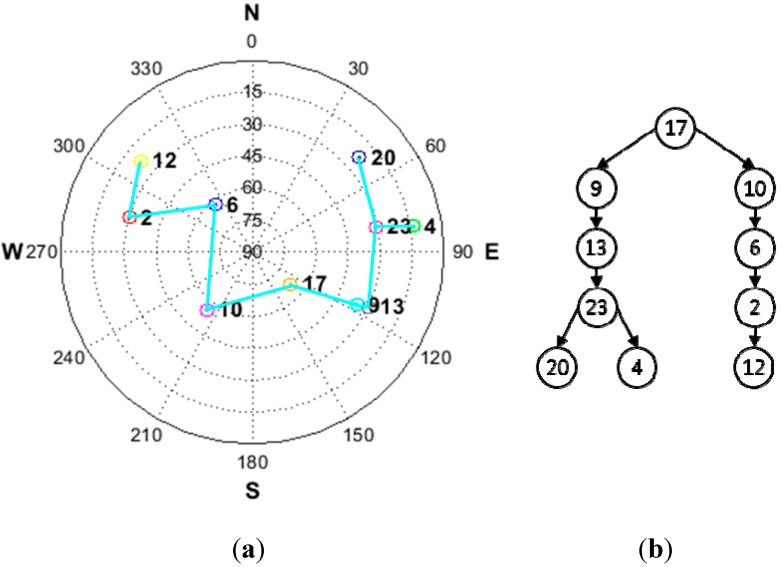
(**a**) Satellite geometry for simulation; (**b**) resulting tree structure for simulation.

Figure 8 represents the considered satellite geometry and resulting tree structure for the SD pair. Among the satellites, that having a PRN of 17 is used as the reference satellite for the general SD which has the highest elevation angle.

#### 4.1.2. Comparing Monitoring Value Error Performance

This section presents the results of comparing the monitoring value for a general SD pair and the proposed SD pair.

**Figure 9:**
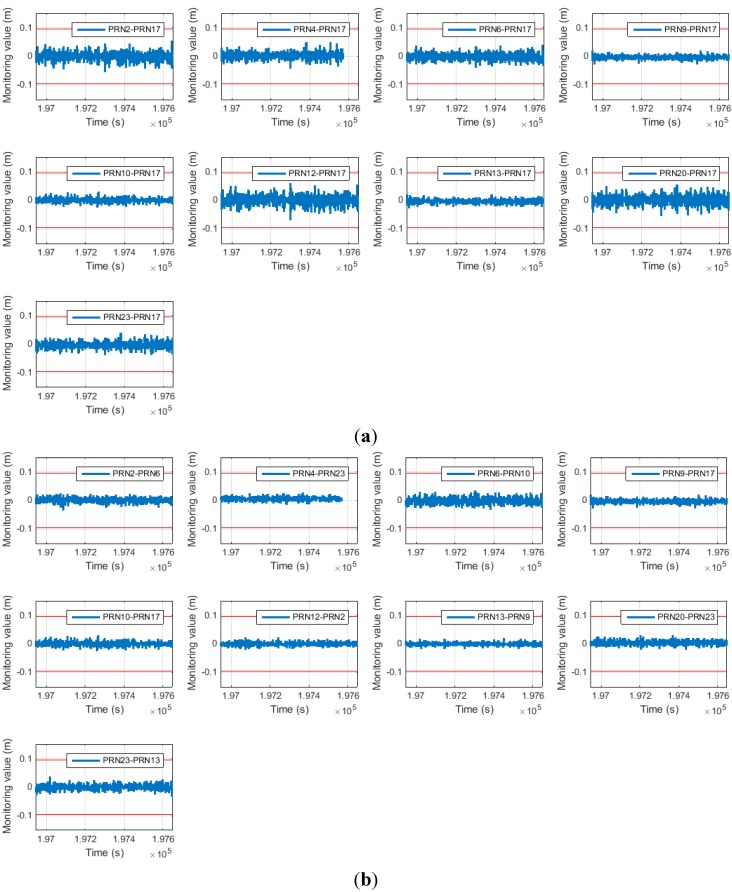
(**a**) Monitoring value error of general SD (above); (**b**) monitoring value error of proposed SD (below).

Figure 9 shows the monitoring value error for both cases. These values can be interconverted in the same manner in Equation (16). The red line represents the half wavelength of the GPS L1 frequency carrier.

It is apparent that the error for the proposed SD pair is much smaller than that for the general SD pair except for satellites 9 and 10. This is because the reference satellites for satellites 9 and 10 are the same as those for the general SD pair. This result is quantitatively summarized in Table 3.

**Table 3:** Standard deviation of monitoring value.

Satellite PRN	σ_*M*_ (General SD) Unit: m	σ_*M*_ (Proposed SD) Unit: m	Error Reduction Unit: %
2	0.0159	0.0093	41.13
4	0.0131	0.0070	46.63
6	0.0130	0.0121	7.03
9	0.0074	0.0074	0
10	0.0082	0.0082	0
12	0.0181	0.0071	60.76
13	0.0081	0.0060	26.17
20	0.0174	0.0083	52.15
23	0.0123	0.0095	23.02

Table 3 shows the standard deviation for the monitoring value errors, which is greatly reduced. According to Figure 5, the decrease in the monitoring value standard deviation directly improves the cycle slip false alarm and miss detection probabilities. In this study, the threshold was designed to attain a miss detection probability of 6.3342 × 10^−5^. Therefore, by reducing the monitoring value variance, the false alarm probabilities are improved. This is summarized in Table 4 (in the table, any probability of less than 1 × 10^−20^ is entered as 0).

**Table 4:** Cycle slip false alarm probability.

Satellite PRN	*P_FA_* (General SD)	*P_FA_* (Proposed SD)
2	2.9127 × 10^−5^	3.700 × 10^−19^
4	1.5366 × 10^−12^	0
6	3.1072 × 10^−6^	3.0773 × 10^−6^
9	0	0
10	1.8400 × 10^−18^	1.8400 × 10^−18^
12	3.7533 × 10^−4^	0
13	0	0
20	8.1364 × 10^−5^	0
23	6.6705 × 10^−11^	9.0578 × 10^−12^

#### 4.1.3. Cycle Slip Occurrence Simulation

To verify the cycle slip detection performance, the generated cycle slips are inserted into the carrier phase. The cycle slips are generated through the scenario described in Table 5.

**Figure 10:**
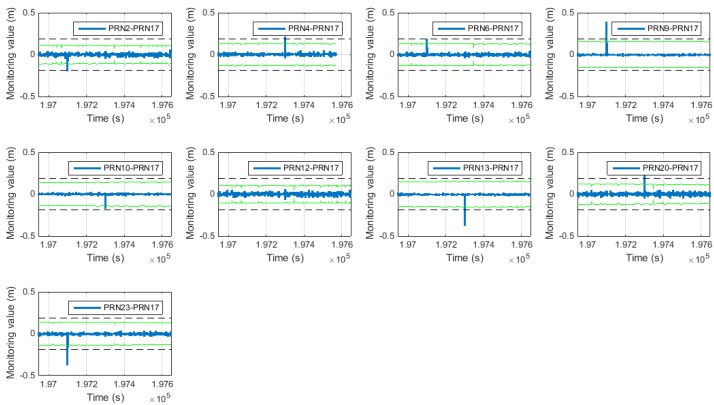

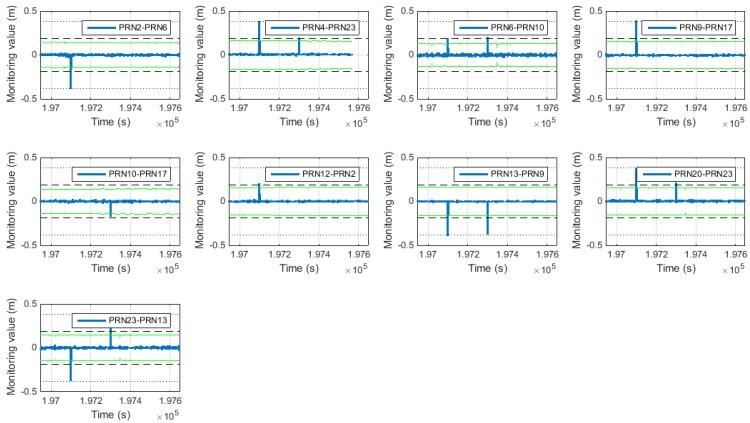
(**a**) Cycle slip detection result of general SD (above); (**b**) Cycle slip detection result of proposed SD (below).

Figure 10 shows the cycle slip detection results for the general SD pair and the proposed SD pair. In Figure 10, the green line represents the cycle slip threshold as determined by using the covariance of the TDCP/INS filter, which varies according to the change in the covariance. The dashed line represents the wavelength of the GPS L1 carrier, which is the same as that for one-cycle slip. The dotted line represents the value equal to double this value.

**Table 5:** Inserted cycle slips.

Time Unit: s	PRN									
	2	4	6	9	10	12	13	17	20	23
197,100	−1		1	2						−2
197,300		1			−1		−2		1	

The cycle slip detection results are summarized in Table 6 and Table 7. All of the cycle slips are correctly detected using both methods. Furthermore, the cycle slip based on the general SD pair can be reconstructed from the cycle slip based on the proposed SD pair, by using the matrix *T* in Equation (38) and the relationship in Equation (16). If we convert the result in Table 7 into the general SD pair by using *T*, we can get the same results as those shown in Table 6. During the data processing, no false alarm occurs, in both cases.

**Table 6:** Detected cycle slips (general SD).

Time Unit: s	PRN								
	2–17	4–17	6–17	9–17	10–17	12–17	13–17	20–17	23–17
197,100	−1		1	2					−2
197,300		1			−1		−2	1	

**Table 7:** Detected cycle slips (proposed SD).

Time Unit: s	PRN									
	2–6	4–23	6–10	9–17	10–17	12–2	13–9	20–23	23–13	
197,100	−2	2	1	2		1	−2	2	−2	
197,300		1	1		−1		−2	1	2	

(38) $$T=\left[\begin{array}{ccccccccc} 1 & 0 & 1 & 0 & 1 & 0 & 0 & 0 & 0\\ 0 & 1 & 0 & 1 & 0 & 0 & 1 & 0 & 1\\ 0 & 0 & 1 & 0 & 1 & 0 & 0 & 0 & 0\\ 0 & 0 & 0 & 1 & 0 & 0 & 0 & 0 & 0\\ 0 & 0 & 0 & 0 & 1 & 0 & 0 & 0 & 0\\ 1 & 0 & 1 & 0 & 1 & 1 & 0 & 0 & 0\\ 0 & 0 & 0 & 1 & 0 & 0 & 1 & 0 & 0\\ 0 & 0 & 0 & 1 & 0 & 0 & 1 & 1 & 1\\ 0 & 0 & 0 & 1 & 0 & 0 & 1 & 0 & 1 \end{array}\right]$$

### 4.2. Experimental Results

#### 4.2.1. Experimental Environments

**Figure 11:**
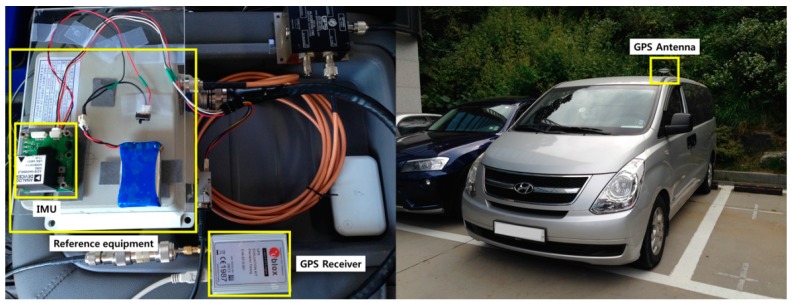
Hardware setup.

For a more practical test of the proposed algorithm, we conducted a static test and a vehicle-based experiment. The static test was conducted in building 301 at Seoul National University. The dynamic experiment was conducted in the parking lot of Seoul Grand Park in September 2014. Figure 11 shows the hardware setup used in the experiment.

A u-blox LEA-6T (u-blox AG, Zürcherstrasse, Switzerland) was used as the GPS receiver and an ADIS16365 IMU (Analog Devices Inc., Norwood, MA, USA), which is of MEMS grade, was used as the inertial sensor. The GPS output rate was set to 1 Hz and the IMU output rate was set to 100 Hz. Raw GPS data and IMU data was collected using a laptop. The GPS antenna was attached to the roof of the vehicle. A SPAN-CPT system (NovAtel Inc., Calgary, Canada) and Waypoint post-processing software (NovAtel Inc.) were used to calculate the true position. This true position was used to compute the true cycle slip occurrence of the u-blox LEA-6T carrier phase measurement. The obtained reference solution is accurate to within 2 cm, which is sufficient to evaluate the true cycle slip occurrence. During the experiment, cycle slip did not occur.

**Figure 12:**
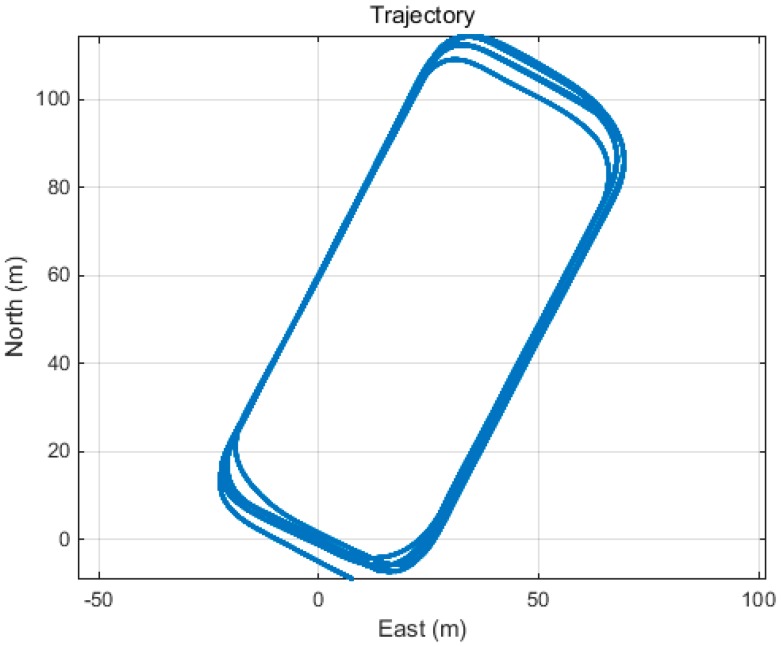
Experimental trajectory.

**Figure 13:**
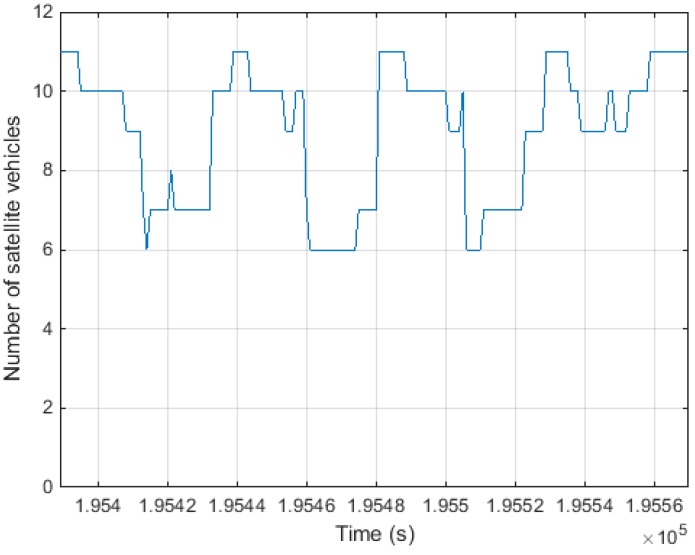
Number of visible satellites.

Figure 12 shows the experimental trajectory. Figure 13 shows the number of satellites during the experiment. Between 6 and 11 satellites are visible. The mask angle for the satellite elevation was fixed to 10°.

**Figure 14:**
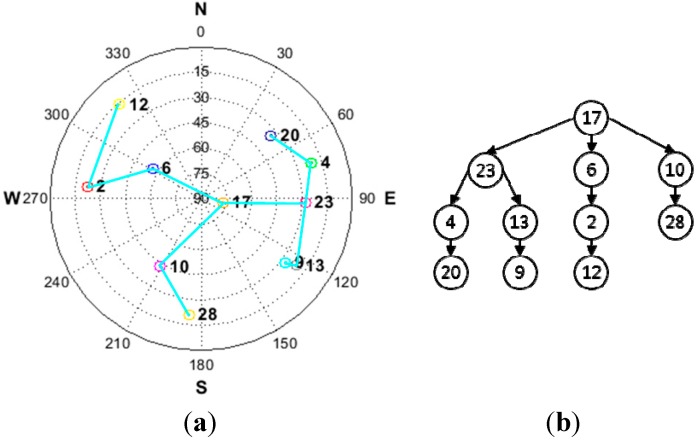
(**a**) Satellite geometry for experiment; (**b**) resulting tree structure.

Figure 14 shows the sky plot and the resulting SD pair when all of the satellites are visible.

**Figure 15:**
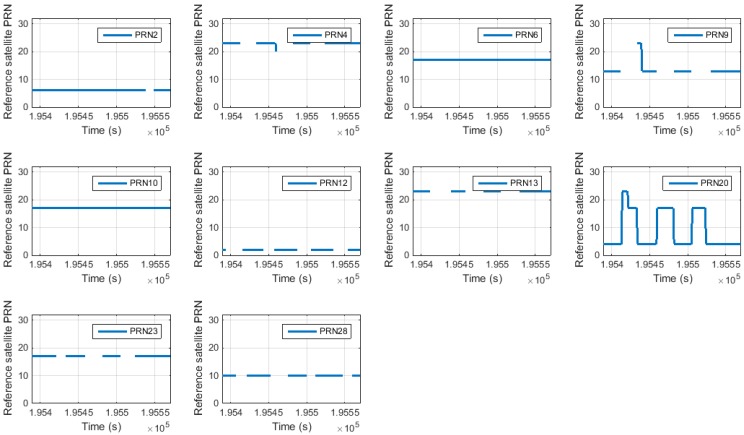
Proposed reference satellite PRN for each satellite.

According to the change in the satellite geometry, the SD pair was automatically changed according to the SD pair construction algorithm. Figure 15 shows the reference satellite selection results as determined using the proposed algorithm.

#### 4.2.2. Analysis of Error Sources of Monitoring Value

**Figure 16:**
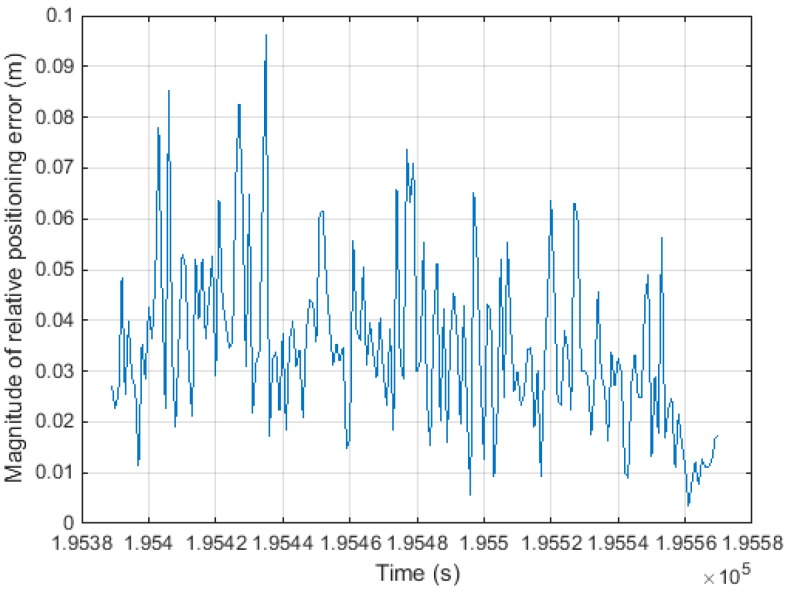
Magnitude of relative positioning error (dynamic test).

**Figure 17:**
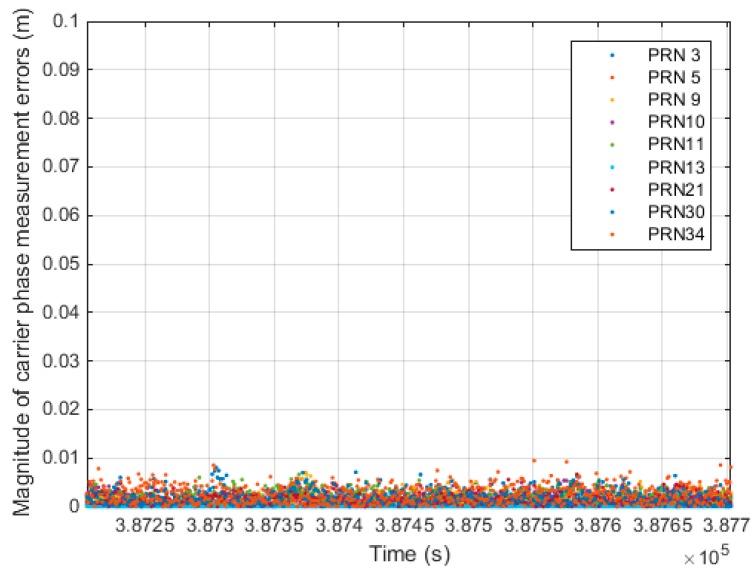
Magnitude of carrier phase measurement errors (static test).

Figure 16 shows the magnitude of the estimated relative positioning error, $\left|\text{δ}{\text{Δ}}_{t}{\vec{r}}_{u}\right|$, as computed from the dynamic test. Figure 17 shows the magnitude of the carrier phase measurement error, |∇jiΔtε|, as calculated from the static test. For the static test, we knew the relative position vector between the epochs and the zero vector. Therefore, the only error source of the monitoring value is the term related to the carrier phase measurement.

By comparing Figure 16 and Figure 17, it can be seen that the dominant error source for the monitoring value is the relative positioning error rather than the carrier phase measurement error. Thus, this confirms the assumptions made during the derivation of Equation (10).

#### 4.2.3. Comparing Monitoring Value Error Performance

**Figure 18:**
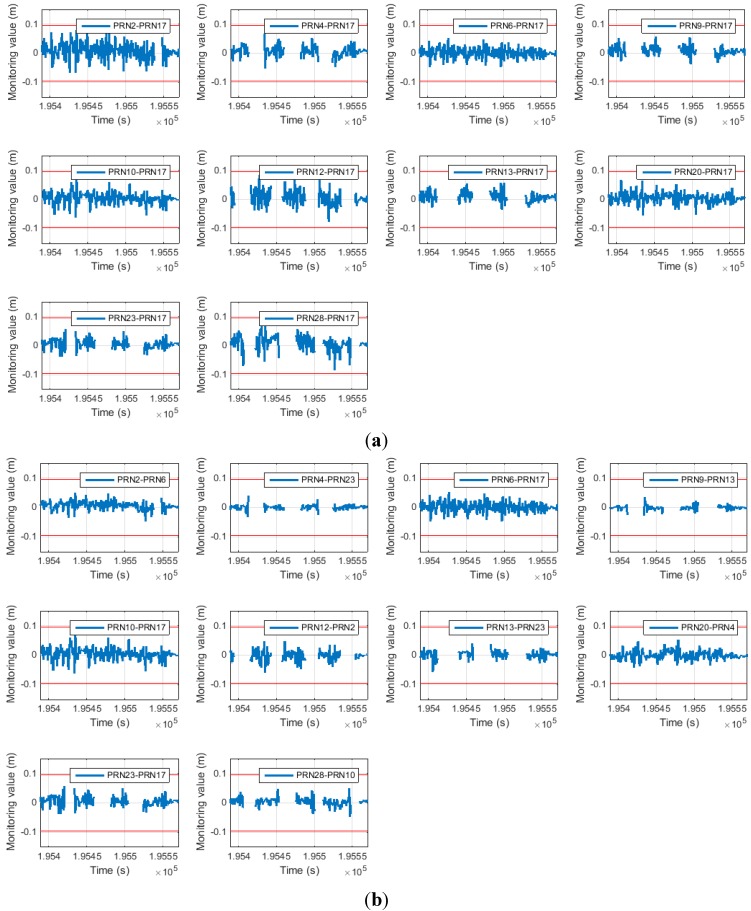
(**a**) Monitoring value error of general SD (above); (**b**) monitoring value error of proposed SD (below).

Figure 18 shows the monitoring value errors for the general SD pair and proposed SD pair. In the same way as for the simulation result, the monitoring value error was reduced for the proposed SD pair.

The monitoring value standard deviation is summarized in Table 8 for both cases. Apart from those satellites, which use the same reference satellite as the general satellite pair, the standard deviations of the monitoring value error decrease, in the same way as in the simulation results.

**Table 8:** Standard deviation of monitoring value.

Satellite PRN	σ_*M*_ (General SD) Unit: m	σ_*M*_ (Proposed SD) Unit: m	Error Reduction Unit: %
2	0.0303	0.0153	49.67
4	0.0194	0.0094	51.71
6	0.0187	0.0187	0
9	0.0173	0.0088	49.29
10	0.0210	0.0210	0
12	0.0293	0.0191	34.87
13	0.0183	0.0148	19.01
20	0.0193	0.0158	18.25
23	0.0179	0.0179	0
28	0.0278	0.0155	44.37

Accordingly, the false alarm probabilities are also improved as shown in Table 9. The cycle slip detection threshold is determined by applying the same requirement as the simulation case.

**Table 9:** Cycle slip false alarm probability.

Satellite PRN	*P_FA_* (General SD)	*P_FA_* (Proposed SD)
2	2.1643 × 10^−2^	2.4368 × 10^−14^
4	4.5633 × 10^−6^	0
6	5.4371 × 10^−7^	5.4371 × 10^−7^
9	6.3644 × 10^−8^	2.4400 × 10^−18^
10	7.2717 × 10^−6^	7.2717 × 10^−6^
12	2.0212 × 10^−2^	2.8827 × 10^−10^
13	2.8758 × 10^−7^	4.8800 × 10^−18^
20	1.3231 × 10^−6^	3.2823 × 10^−7^
23	6.5343 × 10^−9^	6.5343 × 10^−9^
28	1.1157 × 10^−3^	2.4400 × 10^−18^

#### 4.2.4. Cycle Slip Occurrence Simulation

Because no cycle slip occurred during the experiment, we simulated cycle slip occurrence by inserting generated cycle slips into the carrier phase measurement. Table 10 describes the cycle slip occurrence scenario.

**Table 10:** Inserted cycle slips.

Time Unit: s	PRN										
	2	4	6	9	10	12	13	17	20	23	28
195,441	1				−1	−1					−2
195,475		1		−3			−1			1	

**Figure 19:**
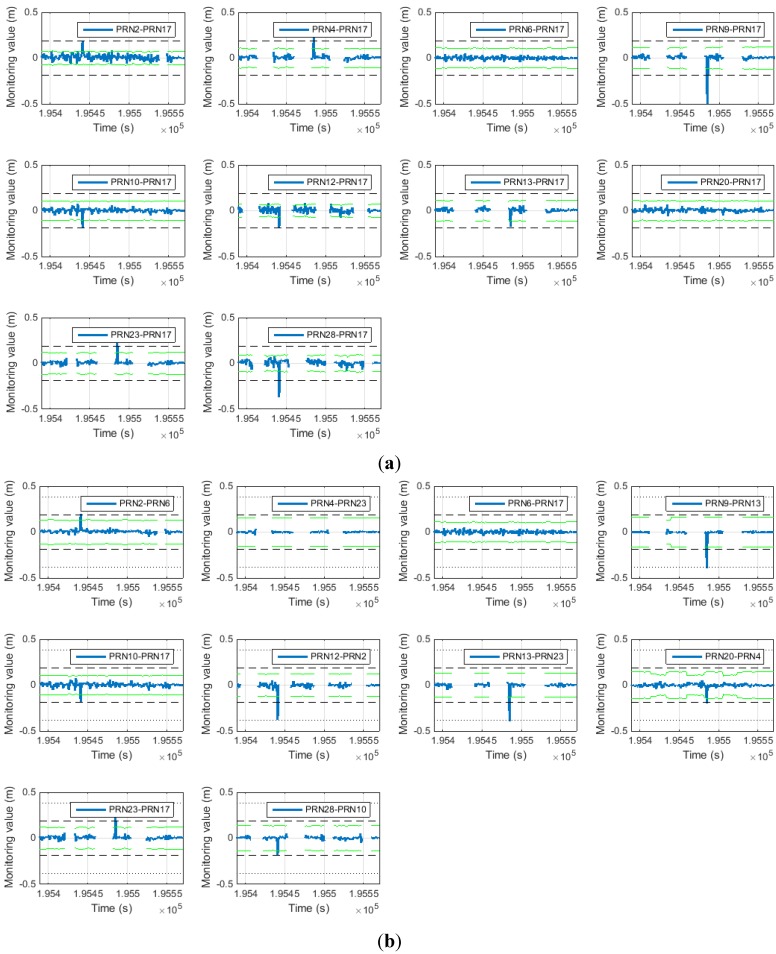
(**a**) Cycle slip detection result of general SD (above); (**b**) cycle slip detection result of proposed SD (below).

Figure 19 shows the cycle slip detection results. All of the cycle slips were correctly detected. In Figure 19, for the satellite with PRN 20, the detection threshold was found to vary periodically. This occurred as a result of specific satellites disappearing during the experiment, causing the reference satellite for PRN 20 to change as shown in Figure 15. As a result, the detection threshold varied accordingly. The variation in the detection threshold was normal.

Table 11 and Table 12 list the detected cycle slips for the general SD pair and the proposed SD pair. All cycle slips are correctly detected.

**Table 11:** Detected cycle slips (general SD).

Time Unit: s	PRN									
	2–17	4–17	6–17	9–17	10–17	12–17	13–17	20–17	23–17	28–17
195,441	1				−1	−1				−2
195,475		1		−3			−1		1	

**Table 12:** Detected cycle slips (proposed SD).

Time Unit: s	PRN									
	2–6	4–23	6–17	9–13	10–17	12–2	13–23	20–4	23–17	28–10
195,441	1				−1	−2				−1
195,475				−2			−2	−1	1	

Equation (39) is the transformation matrix:

(39) $$T=\left[\begin{array}{cccccccccc} 1 & 0 & 1 & 0 & 0 & 0 & 0 & 0 & 0 & 0\\ 0 & 1 & 0 & 0 & 0 & 0 & 0 & 0 & 1 & 0\\ 0 & 0 & 1 & 0 & 0 & 0 & 0 & 0 & 0 & 0\\ 0 & 0 & 0 & 1 & 0 & 0 & 1 & 0 & 1 & 0\\ 0 & 0 & 0 & 0 & 1 & 0 & 0 & 0 & 0 & 0\\ 1 & 0 & 1 & 0 & 0 & 1 & 0 & 0 & 0 & 0\\ 0 & 0 & 0 & 0 & 0 & 0 & 1 & 0 & 1 & 0\\ 0 & 1 & 0 & 0 & 0 & 0 & 0 & 1 & 1 & 0\\ 0 & 0 & 0 & 0 & 0 & 0 & 0 & 0 & 1 & 0\\ 0 & 0 & 0 & 0 & 1 & 0 & 0 & 0 & 0 & 1 \end{array}\right]$$

If we convert the results in Table 12 to a general SD pair by applying Equations (39), we can get the same results as those listed Table 11.

Table 13 shows the false alarm occurrence during the data processing.

**Table 13:** False alarm rates.

Method	PRN									
	2	4	6	9	10	12	13	20	23	28
General SD	7	0	0	0	0	5	0	0	0	2
Proposed SD	0	0	0	0	0	0	0	0	0	0

In the results obtained with the general SD method, 14 false alarms occur, unlike with the proposed SD method in which no false alarm occurs.

Practically, the detected cycle slip can be treated in either of two ways. One is an isolation and the other is a repair. With the isolation, a false alarm causes a decrease in the number of satellites that can be used for positioning. This can lead to a decrease in the positioning accuracy by increasing the DOP. In the worst case, the positioning cannot be performed if the number of usable satellites is insufficient. For the repair, a false alarm also degrades the positioning accuracy with the wrong cycle slip repair. Consequentially, the proposed SD method offers greater capability than the general SD method.

#### 4.2.5. Computational Load Analysis

To analyze the computational load of the proposed algorithm, the total computation time with the general and proposed methods is computed. Table 14 lists the calculation results.

**Table 14:** Computational load comparison.

Computation Time Unit: s		Increase Rate Unit: %
General SD	Proposed SD	
30.5	32	0.05

During the data processing, SD pair update is conducted 40 times. As a result, the computation time increases by 0.05%. Therefore, we can conclude that the computational load of the proposed algorithm is permissible.

### 4.3. Summary of Simulation and Experimental Results

In summary, the proposed algorithm reduced the monitoring value error caused by the INS positioning error. The reduction rate was about 10% to 60%. Accordingly, the cycle slip false alarm probabilities also improved. In cycle slip occurrence simulation, a minimum of one-cycle slip is possible for both the simulation and experiment. Furthermore, by using the transformation matrix, the cycle slips based on the general SD pair can be easily reconstructed.

## 5. Conclusions

This paper proposes an inertial-aided cycle slip detection method that takes the satellite geometry into account. To precisely detect the cycle slip of a single-frequency receiver, INS is utilized. In addition, unlike existing inertial-aided cycle slip detection algorithms, the satellite geometry is used to attain accurate range estimation. By constructing an SD pair which minimizes the projection coefficient of the INS position estimation error to the range domain, we can improve the cycle slip detection performance without an inertial sensor upgrade. We also introduce a tree structure to attain an independent SD pair, which also enables conversion between the proposed SD pair and any other SD pair with no loss of information. This means that the proposed algorithm can be applied to the carrier-phase-based positioning algorithm using any SD pair, including the general SD pair. Regarding the computational load, the update interval for the SD pair can be anything up to a few minutes in duration, since the satellite geometry changes only slowly. Therefore, this algorithm offers a computational advantage.

A simulation and land-vehicle-based experiment were conducted to investigate the performance of the proposed algorithm. In both cases, the proposed algorithm effectively improves the cycle slip detection performance. With a MEMS IMU, one-cycle slip can be successfully detected by using single-frequency GPS receiver measurements when there are more than four visible satellites. As a result, the cycle slip detection performance of the proposed algorithm is verified.
